# Development of an Ozone-Assisted Sample Preparation Method for the Determination of Cu and Zn in Rice Samples

**DOI:** 10.1155/2021/5586227

**Published:** 2021-07-19

**Authors:** Mariela Pistón, Ignacio Machado, Esteban Rodríguez-Arce, Isabel Dol

**Affiliations:** Grupo de Análisis de Elementos Traza y Desarrollo de Estrategias Simples para Preparación de Muestras (GATPREM). Analytical Chemistry, Faculty of Chemistry, Universidad de La República, Montevideo, Uruguay

## Abstract

A green analytical method for the determination of Cu and Zn in rice samples was developed. This method was based on an ozone-assisted extraction (OAE) in diluted acid media. A novel closed system was designed for this purpose that allowed four simultaneous sample treatments being safe for the laboratory environment. The method consisted in 0.5 g of the sample, 15 minutes of ozonation, and 3 minutes of centrifugation. The obtained supernatant was ready for Cu and Zn determinations by flame atomic absorption spectrometry. Detection limits were 0.20 and 0.08 mg kg^−1^ for Cu and Zn, respectively, with a precision (RSD) better than 5% for both elements. A certified reference material of rice flour was analyzed for trueness evaluation, and the mean recoveries (%) were 100.4 (Cu) and 95.9 (Zn). Several commercial rice samples were analyzed using this method, and the results were compared with those obtained using traditional microwave-assisted digestion (MAE). Both methods yielded comparable results. Cu and Zn levels were in accordance with reported values in other regions. The OAE resulted to be simple and economical and with results equivalent with those obtained using traditional sample preparation procedures as MAE with the advantage of being in good agreement with the principles of green analytical chemistry.

## 1. Introduction

Rice (*Oryza sativa* L.) is a massively consumed food item in the world. Besides being an immediate source of energy, this cereal has several health benefits. Both white rice and brown rice contain unique nutritional value and are gluten-free foods; therefore, they can be easily included in the diet of people suffering from celiac disease.

The determination of the micronutrient content such as copper (Cu) and zinc (Zn) in different types of rice is not only important to know about its nutritional value but also to characterize the cereal from the point of view of other characteristics such as its genotype [1]. These elements are also of interest to monitor anthropogenic activities that can generate contamination in food [2, 3].

Analytical determinations of trace elements in food based on standard methods, which are still in force, are permanently revised and tend to decrease sample processing. Despite this, most analytical methods are still based on acid digestions or dry ashing that take a long time, have many stages, and consume large amounts of dangerous reagents (mainly mineral acids to oxidize the organic matter to extract the trace elements) [4]. More recently, the incorporation of the microwave-assisted treatments (MAE) and the use of diverse strategies to reduce the acid consumption became more efficient sample preparation methods [5, 6].

Equipment for the detection and quantification of trace elements has evolved rapidly, but it is useless to have instruments according to the state-of-the-art detection techniques if sample preparation is not performed correctly, efficiently, in clean areas, trying to minimize intermediate stages of manipulation to reduce errors and costs.

The 12 principles of green chemistry (GC) are well established since decades and have progressed in parallel with the principle of green analytical chemistry (GAC). Initially, of the 12 principles of GC, only a few were applicable in the development of new analytical methods, and other aspects related to the figures of merit were not considered [7, 8]. This generated the need to adapt analytical methods, and those used for food analysis should not be alien to this. MAE has been used since the 1980s in trace element analysis, but alternatives that improve these methods by performing them under milder conditions have become increasingly necessary. More recently, the use of ultrasound-mediated extractions has gained interest, and there are already numerous publications demonstrating that they are efficient [9–13], but the use of ozone-mediated extraction has been little explored.

One of the first steps in sample preparation before analytical determinations is the extraction of the elements quantitatively from the matrix. In this respect, oxidation processes that involve the *in situ* generation of oxidant chemical species have been reported as an effective strategy to accelerate the degradation process of organic matter, at room temperature, in several matrices (wine, effluents, and green vegetables) [6, 14, 15].

These processes, called AOPs (advanced oxidation processes), can use ozone gas. Ozone (O_3_) is a powerful oxidizing agent, which has an oxidation potential of 2.07 V. It is naturally formed in the atmosphere and was discovered by the European researcher C. F. Schönbein in 1939. It is a colorless gas with a strong characteristic odor that has been described as “something similar to the smell of fresh air after a storm.” Under standard conditions of temperature and pressure is an unstable gas that rapidly transforms into molecular oxygen [16]. Ozone can also oxidize heavy metals. The oxidizing capacity is used to remove organic matter and heavy metals that are otherwise difficult to remove from, e.g., industrial waters or effluents [17, 18]. It was also used to remove organic pollutants present in water and pollutants emerging in effluents from the pharmaceutical industry taking advantage of this oxidizing capacity to degrade organic compounds [19].

Based on the properties of ozone, a rapid sample preparation procedure was developed and validated with the use of dilute acid to extract Cu and Zn from rice samples. Results were equivalent to those obtained using traditional microwave-assisted procedures (MAE) and with adequate figures of merit in addition to the advantages of the reduction of waste generation and in better agreement with the principles of green analytical chemistry.

## 2. Materials and Methods

Calibration standards were prepared from a 1000 mg L^−1^ commercial solution containing Cu and Zn (Merck, Darmstadt, Germany). All the materials were decontaminated using 10% (v/v) HNO_3_ (p.a., Merck, Darmstadt, Germany). Purified water (resistivity: 18.2 MΩ cm) was obtained from a Millipore water purification system. Nitric acid 67% (Merck, Darmstadt, Germany). An aqueous silicone emulsion (Antifoam B, Sigma-Aldrich) was used as an antifoam agent.

### 2.1. Samples

The determinations of Cu and Zn were carried out in seven samples of Uruguayan rice available in the national market, five of white rice (brands named as *A*, *D*, *E*, *F*, and *G*), one of brown rice (brand *A*), and one parboiled (brand *C*). This selection represents the brands with the highest consumption in the country and exporters of rice from Uruguay. For validation, a certified reference material (CRM, NIST 1568a rice flour) was analyzed.

The samples were ground with a blade mill for 5 minutes. The obtained flour (200 *µ*m particle size) was subjected to the ozonation treatment and a total digestion by microwave-assisted digestion. All treatments were carried out in triplicate.

### 2.2. Sample Preparation

#### 2.2.1. Ozone-Assisted Extraction (OAE)

The proposed method consisted of weighing 0.5 g of rice flour that was placed in a glass container (midget impinger 25 mL tube), 10.00 mL of 3.5 mol L^−1^ HNO_3_ was added, it was homogenized, and two drops of antifoam silicone were added; this suspension was ozonized for 15 minutes.

**Figure 1:**
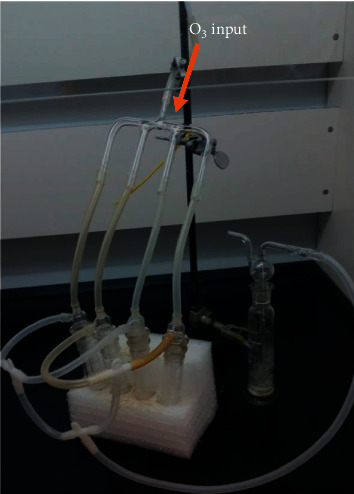
Device for the OAE. Four commercial glass (borosilicate 3.3, Pyrex^®^) midget impinger (25 mL) tubes with fritted nozzles allow to analyze a sample by triplicate and a blank simultaneously. At the bottom, a flask (trap) containing Na_2_S_2_O_5_ solution is placed to neutralize the excess of ozone gas and close the system.

Figure 1 shows the developed system for ozonation. This device used for OAE allowed the treatment of each sample in triplicate and with a reference blank simultaneously. Ozone gas was generated from oxygen with an ozone generator (OZOX—OG 75-A, Montevideo, Uruguay) with a yield of 1.6 g h^−1^, with an ozone-enriched oxygen flow of 7 L min^−1^. The main gas input was divided into four individual tubes and passes through a porous glass membrane situated at the end of each one (pore size: 200 *μ*m) into the solution containing the sample (or blank) and diluted acid.

The system used silicone tubes for the unions, and at the end, a ﬂask was placed with a Na_2_S_2_O_5_ solution, 5% (w/v). Thus, the excess of ozone gas generated in the process does not generate contamination of the environment in the laboratory. With this design, the system remains totally closed and safe.

After the ozonation process, the suspension obtained was centrifuged for 3 minutes at 28,000 *g* (UNICO, PowerSpin^TM^, DX Centrifuge, NY, USA), and the supernatant was directly used to perform Cu and Zn measurements. The process was carried out at room temperature.

Under the experimental conditions of this method, the ozone concentration in solution was 21 mg L^−1^. This concentration was obtained following the procedure previously reported by our research group by titration [15].

#### 2.2.2. Microwave-Assisted Extraction (MAE)

For comparison purposes, a microwave-assisted extraction (MAE) was performed. In this case, 0.5 g of each rice flour sample was placed in the reaction vessels of the equipment (CEM MARS 6 equipped with EasyPrep Plus^®^ vessels) with 10.00 mL of HNO_3_ 1 : 1 (acid concentrated: ultrapure water). The program used a power between 400 and 1800 W, a heating ramp up to 200°C for 15 minutes, and a holding time of 10 minutes at 200°C and finally was cooled down for 15 minutes before opening the vessels. A limpid solution was obtained after digestion that was transferred to a 15 mL conic plastic tube (Falcon™), and the volume of 10.00 g was completed gravimetrically. Then, the subsequent Cu and Zn determinations were carried out. Each sample was analyzed by triplicate, and reagent blanks were also run.

### 2.3. Analytical Determinations

The determinations of Cu and Zn were carried out by means of flame atomic absorption spectroscopy (FAAS) using a PerkinElmer AAnalyst 200 spectrometer (PerkinElmer, USA), operated at 213.86 nm (Zn) and 324.75 nm (Cu), respectively. Hollow cathode lamps (PerkinElmer, USA) were operated as recommended by the manufacturer. A 10 cm burner was used, the flame was composed of air/acetylene (10.0 and 2.5 L min^−1^, respectively), and no background correction was used.

Five-point calibration curves of 0.1, 0.5, 1.0, 2.0, and 5.0 mg L^−1^ and 0.1, 0.25, 0.75, and 1.0 mg L^−1^ for Cu and Zn, respectively, were prepared by appropriate dilution from the 1000 mg L^−1^ stock for the determinations using the OAE and the MAE methods. In the case of the OAE, aqueous standards were used, while for the MAE method, it was necessary to use HNO_3_ 1 : 1 in ultrapure water to avoid matrix effect to prepare the calibration solutions.

Method validation was performed according to the recommendations of the Eurachem Guide [20]. The main figures of merit assessed were linearity, detection, quantification limits (LOD and LOQ), precision, and trueness. A CRM of rice flour (NIST 1568a rice flour) was analyzed to guarantee accuracy of the developed method.

## 3. Results and Discussion

### 3.1. Validation

In previous works, multivariate experimental designs were performed in our laboratory to obtain optimal ozonation time and nitric acid concentration for green vegetables (spinach and artichoke) [6]. Based on the experience, experimental conditions close to those reported were tested (ozonation time: 10, 15, and 20 minutes and nitric acid: 2, 3.5, and 5 mol L^−1^), and the best results in terms of accuracy and precision were obtained with 15 min of ozone treatment and HNO_3_ 3.5 mol L^−1^. According to Machado et al., in the case of green vegetables, 10 minutes of ozonation were adequate; rice required 15 minutes. This can be attributed to the complexity and particular characteristics of this matrix (high content of carbohydrates).

Validation was carried out following the Eurachem Guide; the main figures of merit are evaluated and summarized in Table 1 for the OAE and MAE procedures. LOD and LOQ were obtained using the 3*s*/*m* and 10*s*/*m* criteria, with *s* being the standard deviation of the blank's signals and *m* being the slope of the calibration curves. The blanks were subjected to the same treatment as the samples, as it can be observed in Figure 1; this treatment was simultaneous to the triplicate analysis of a sample.

Calibration curves presented *R*^2^ > 0.998 for both elements, but it is important to highlight that, for the OAE method, aqueous standards were used, while for the MAE, standards must be prepared in HNO_3_ 1 : 1 (approx. 7 mol L^−1^). The use of MAE frequently requires a calibration where the characteristics of the sample must be equalized to avoid calibration errors. The use of HNO_3_ in high concentration causes deterioration of the equipment because of corrosion in addition to being dangerous.

For trueness evaluation, a CRM of rice flour was analyzed, and then the entire process was repeated six times (triplicate of the CRM and a blank each time). Copper and Zn mean recoveries were of 100.4 and 95.9%, respectively. Precision expressed as RSD% for each sample in all cases was better than 5%.

Comparing the figures of merit, both procedures are adequate for this purpose. Results demonstrate that the proposed method (OAE) is accurate and can be used as an alternative for the analysis of rice samples and in better agreement with the principles of green analytical chemistry than the MAE method.

### 3.2. Analysis of Rice Samples

Once the OAE was validated, it was applied to analyze rice commercial samples. Seven different brands were selected in the local markets; one of them was brown rice and another was parboiled.

The results obtained using the new method were compared with those obtained with a MAE. As it can be observed in Table 2, the results for both procedures are equivalent. This means that the OAE is also reliable compared with traditional sample preparation methods.

The trace element content in rice is highly dependent on the irrigation conditions and the paddy soil. Several authors studied the trace element content of rice to provide a profile to discriminate different genotypes or geographical origin [1, 21]. Lange et al. reported that the Zn content was one of the main variables that allowed to discriminate the rice origin of samples from Brazil; in their work, samples were taken from a nearby geographical zone to Uruguayan rice crops, and our results were in accordance with their results [22]. Despite the dependence of the trace element composition and the geographical zone, reported ranges of Cu and Zn levels are wide, and these results were also in good agreement with those found in polished rice from China (mean value for Cu: 2.7 mg kg^−1^ and 20.9 mg kg^−1^ for Zn) [1, 3, 23].

The sample “brand *C*” was parboiled; there was no declaration of fortification in the label. Cu level was similar to other polished rice samples, but the Zn content was significantly lower. Cooking processes can affect mineral concentrations in food, particularly Zn content, as reported by Biswas et al. Thus, the industrial boiling process can be exploited for fortification with micronutrients [24, 25]. Therefore, our results confirm the importance of the fortification of rice with Zn before commercialization during the parboiled process since after cooking, an important proportion of this essential element is lost.

### 3.3. Green Analytical Chemistry (GAC)

The developed method complies with several of the 12 principles of GAC, satisfactory, and improves sample preparation conditions regarding MAE. If the complete analytical process is considered, once the method has been validated, calibration can be carried out only with one or two standards, for routine analysis, prepared in the aqueous medium, for the extraction, more dilute acid is used compared with MAE, and the time to obtain four treatments is 15 minutes and 3 minutes of centrifugation.

Ozone production is a low-cost technology and is carried out using the corona discharge method from oxygen, and the process is carried out at room temperature. In addition, it should be considered that these improvements do not go to the detriment of the figures of merit since adequate precision and accuracy were achieved, and in this sense, the proposed method also complies with specific principles of GAC regarding figures of merit. Thus, the OAE can be considered, without doubts, greener than the traditional MAE for the stated objective.

## 4. Conclusions

A novel device for the ozone-assisted extraction of Cu and Zn from rice samples was developed. This method allowed to perform four treatments simultaneously using diluted acid with 15 minutes of ozonation in a closed system. The analytical determinations of Cu and Zn were performed directly on the supernatant after centrifugation by means of flame atomic spectrometry. The proposed method was validated, resulting in a simple and reliable alternative to microwave-assisted sample treatment technology.

Commercial samples of Uruguayan rice were analyzed, and the results were in accordance with other previously reported results from other regions. Parboiled rice presented significantly less Zn content than polished samples which are not cooked.

This analytical method resulted in good agreement with the principles of green analytical chemistry and can be proposed as a new alternative to explore for trace element analysis in foodstuffs.

## Figures and Tables

**Table 1:** Figures of merit for the OAE and MAE methods.

Parameter	Cu		Zn	
	OAE	MAE	OAE	MAE
Linear range (*µ*g L^−1^)	Up to 5.0 mg L^−1^		Up to 1.0 mg L^−1^	
LOD (mg kg^−1^, *n* = 6)	0.20	0.18	0.08	0.10
LOQ (mg kg^−1^, *n* = 6)	0.65	0.60	0.26	0.33
Precision (RSD%, OAE n = 18^*∗*^ and MAE *n* = 10)	3.0	3.1	2.9	3.3
Certified value (mg kg^−1^) (NIST 1568a)	2.4 ± 0.3		19.4 ± 0.5	
Obtained value (mg kg^−1^)	2.41 ± 0.09	2.32 ± 0.06	18.6 ± 0.6	19.8 ± 0.7
Trueness (*R*%^*∗∗*^, OAE *n* = 18^*∗*^ and MAE *n* = 10)	100.4	96.7	95.9	102.1

Results are expressed as mean ± standard deviation. ^*∗*^The replicate number arises from performing the OAE six times (3 simultaneous sample treatments each time plus a blank) considering the device design. ^*∗∗*^Recovery (%) = (obtained value/certified value) × 100.

**Table 2:** Cu and Zn levels in commercial rice samples.

	Cu (mg kg^−1^)		Zn (mg kg^−1^)	
Samples	OAE	MAE	OAE	MAE
Brand *A*	3.36 ± 0.05	3.27 ± 0.04	20.9 ± 0.9	19.6 ± 0.7
Brand *B* (brown)	2.82 ± 0.03	2.77 ± 0.02	29.5 ± 0.9	30.8 ± 0.3
Brand *C* (parboiled)	2.63 ± 0.08	2.52 ± 0.08	7.62 ± 0.91	9.10 ± 0.85
Brand *D*	2.46 ± 0.04	2.42 ± 0.03	17.4 ± 0.8	16.8 ± 0.3
Brand *E*	2.38 ± 0.07	2.31 ± 0.08	15.4 ± 0.5	14.9 ± 0.4
Brand *F*	3.03 ± 0.08	2.97 ± 0.07	21.1 ± 0.7	21.7 ± 0.4
Brand *G*	3.13 ± 0.06	3.07 ± 0.05	16.2 ± 0.5	16.3 ± 0.3

Results are expressed as mean ± standard deviation (*n* = 3), dry basis.

## Data Availability

The data used to support the findings of this study are available at the repository of the Universidad de la República, Uruguay, PhD thesis (http://riquim.fq.edu.uy/items/show/4312).
